# Return to Work Interventions for Cancer Survivors: A Systematic Review and a Methodological Critique

**DOI:** 10.3390/ijerph16081343

**Published:** 2019-04-14

**Authors:** Kristopher Lamore, Thomas Dubois, Ulrike Rothe, Matilde Leonardi, Isabelle Girard, Ulf Manuwald, Soja Nazarov, Fabiola Silvaggi, Erika Guastafierro, Chiara Scaratti, Thierry Breton, Jérôme Foucaud

**Affiliations:** 1 Institut National du Cancer (INCa), 92100 Boulogne-Billancourt, France; kristopher.lamore@gmail.com (K.L.); tdubois@institutcancer.fr (T.D.); igirard@institutcancer.fr (I.G.); tbreton@institutcancer.fr (T.B.); 2 Laboratory of Psychopathology and Health Processes (EA 4057), Paris Descartes University, 92100 Boulogne-Billancourt, France; 3 Health Sciences/Public Health, Faculty of Medicine Carl Gustav Carus, Technische Universität Dresden, 01307 Dresden, Germany; ulrike.rothe@tu-dresden.de (U.R.); ulf.manuwald@tu-dresden.de (U.M.); soja.nazarov@tu-dresden.de (S.N.); 4 Neurologia, Salute Pubblica e Disabilità, FONDAZIONE IRCCS ISTITUTO NEUROLOGICO CARLO BESTA, 20133 Milan, Italy; matilde.leonardi@istituto-besta.it (M.L.); fabiola.silvaggi@istituto-besta.it (F.S.); erika.guastafierro@istituto-besta.it (E.G.); chiara.scaratti@istituto-besta.it (C.S.); 5 Health Education and Practices Laboratory (LEPS EA 3412), Paris 13 University-UFR SMBH, 93017 Bobigny, France

**Keywords:** cancer, intervention, return to work, systematic review, work rehabilitation

## Abstract

Cancer patients are more at risk of being unemployed or having difficulties to return to work (RTW) compared to individuals without health concerns, and is thus a major public health issue. The aim of this systematic review is to identify and describe the interventions developed specifically to help cancer patients to RTW after treatment. Two researchers independently screened the articles for inclusion and Critical Appraisal Skills Program (CASP) checklists were used to assess the methodology of the included studies. Ten manuscripts met the inclusion criteria. The type of studies were three quasi-experimental studies, three longitudinal studies, three randomized controlled trials (RCTs) and a qualitative study. RTW interventions were conducted in or outside the hospital (*n* = 6 and 3 respectively), or both (*n* = 1). Improvements in RTW were only observed in quasi-experimental studies. No improvement in RTW was noted in RCTs, nor in other measures (e.g., quality of life, fatigue). Lack of statistically significant improvement does not necessarily reflect reality, but may be attributed to non-adapted research methods. This systematic review underscores the need for researches in the RTW field to reach a consensus on RTW criteria and their assessment. Recommendations to this effect are suggested.

## 1. Introduction

Cancer is one of the leading causes of death in the world, with 18 million new cases and 9.6 million deaths from the disease in 2018 [1]. The number of people with cancer has been increasing steadily for the last 10 years worldwide, with a 33% increase in the number of cases between 2005 and 2015 [2]. Over the last decade, however, improvements in early detection and the development of novel therapeutic approaches have contributed to an increase in survival rates [3]. In 2018, 43.8 million people were cancer survivors [1], equivalent to the population of Argentina. In the USA, around 15.5 million (5% of the population) are cancer survivors and this number is estimated to exceed 20 million by 2026 [4]. About half of these people are of working age and are able to return to work (RTW) [5,6,7]. RTW can improve cancer survivors’ quality of life [8,9], as it provides a sense of ‘normality’ and a feeling of social belonging [10]. Employment and working conditions are also social determinants of health [5]. Yet, unemployment rate—around 30%—observed in cancer patients [11,12] is up to 10 times higher than in individuals without health concerns in Europe [13], and 8 times higher in North America [14]. This represents a major public health issue, underlying a social inequality, alerting international health agencies and ministries [15,16,17,18].

Unfortunately, to date, RTW after a severe illness is not defined in the scientific literature, for a concept used since the late 80s, recently highlighted by the Australian Government [19]. Researchers assume that RTW is ‘when workers restore their former lifestyle’ [17,18]. 

Several factors impact work ability [19,20,21], such as cognitive impairment (e.g., memory deficits, concentration problems), physical limitations (e.g., functional disability, pain), as well as psychosocial difficulties (e.g., anxiety, depression, fatigue) [11,17,19,20,21]. Cancer patients and survivors often express concerns relating to the workplace (e.g., disclosing their diagnosis), to their work ability, their physical appearance and to negotiating workplace accommodations with employers [20,22,23]. They also express a need to be guided and supported by health care professionals and vocational providers to RTW [24,25,26].

Over the last few decades, interventions have thus been implemented to help cancer survivors to RTW after treatment. One previous review published in 2009 [27] found four articles presenting intervention studies for breast cancer survivors. These interventions were multidimensional and focused on improving physical, psychological and social recovery, with the outcome RTW. In the studies found, 75% to 85% of the participants RTW after rehabilitation. However, the design of these intervention studies does not allow to know whether these results are due to the intervention, as three of the studies did not include a comparison group. A Cochrane review by De Boer et al. [28] found fifteen articles describing randomized controlled trials (RCTs) of RTW interventions in several cancer locations. The results showed that single-dimensional interventions (psycho-educational, medical or physical) compared to multidimensional interventions did not improve RTW. However, the interventions reported in these two systematic reviews were not developed specifically to improve RTW after cancer treatment, though RTW or employment status were evaluated as an outcome of the intervention. 

The aim of this systematic review is to identify and describe intervention studies developed specifically to help cancer patients RTW. Based on the results highlighted, recommendations for designing RTW interventions and to assess RTW are suggested. This study was carried out within the framework of the EU “Chrodis Plus” Joint Action, a 3-year project that involves 42 beneficiaries representing 20 European countries and covers the field of employment and chronic diseases, among other themes. The aim of this Joint Action is to implement good practices for chronic diseases (http://chrodis.eu/).

## 2. Materials and Methods

### 2.1. Search Strategy

Before conducting this systematic review, a search in the Prospero database revealed that, to date and our knowledge, no literature review is currently underway on this subject. To conduct the present systematic review, we followed the guidelines described by the Preferred Reporting Items for Systematic Reviews and Meta-Analysis (PRISMA) [29]. 

A comprehensive search covering the period 1806–2018 (i.e., comprising all the publications available on the databases) was performed in different international databases: PubMed (1809 to March 31, 2018), PsycINFO (1806 to April 2, 2018) and Embase (1947 to April 1, 2018). Our search was limited to original studies published in the English language and in peer-reviewed journals. The research was conducted by UR, based on a list of search terms developed with the research team in line with the research objective (see Box 1). The research equations used on the databases are presented in the Supplementary Material File S1.

Box 1 **Search terms used on the databases.**

cancer

AND

return-to-work OR re-integrating OR back to work OR employment OR employment sector OR sick leave OR absenteeism OR occupational medicine OR occupational health R occupational health services OR disability management OR disability prevention OR employer*

AND

rehabilitation OR rehabilitation program OR training program * OR training tool* OR training OR occupational rehabilitation OR occupational intervention OR workplace intervention OR occupational therapy OR stress management OR work ability

AND

evaluation study OR evaluate* OR effects OR effectiveness OR efficiency OR process OR outcome OR randomized controlled trial OR controlled clinical trial

### 2.2. Inclusion and Exclusion Criteria

Studies using a qualitative, quantitative or mixed design were included if they satisfied the following criteria: (a) describe an intervention to help RTW for cancer patients being treated or after treatment completion; (b) conducted on patients aged 18 and over and diagnosed with cancer (all locations); (c) written in English; (d) published in peer-reviewed journals. Exclusion criteria included reviews, case control studies, protocol studies (as the RTW intervention is described but not evaluated) and studies which were not evaluated/tested or did not aim to RTW.

### 2.3. Study Selection and Data Extraction

All research results have been merged into EndNote X8.2 reference manager. After removal of duplicates, titles and abstracts were screened. A list of potentially eligible articles liking the inclusion criteria was obtained. When in doubt, article full text reading was performed. Full texts of potentially eligible studies were then reviewed independently by two researchers (Jérôme Foucaud and Kristopher Lamore) to establish a final list of eligible studies. Data were then extracted by K.L. and checked for accuracy by J.F. In case of disagreement, the opinion of a third researcher (Thomas Dubois) was requested and a consensus was reached between researchers (Jérôme Foucaud, Kristopher Lamore and Thomas Dubois). To aim at a near exhaustive list, additional studies were searched using the reference list of the selected manuscripts. A description of all studies was first performed. The information collected from all studies were: authors, year, country, study design and methods, intervention (structure and implementation), objective(s), primary and secondary outcomes(s), population (cancer site, age, work status, level of education) and main results. 

### 2.4. Critical Appraisal of Study Quality

Using the relevant version of the Critical Appraisal Skills Programme (CASP) checklists for cohort studies [30], qualitative studies [31] or RCTs [32], a methodological quality appraisal of the included studies was performed independently by two researchers (J.F. and K.L.). When discrepancies appeared, oral discussion of the manuscripts was performed. Briefly, CASP checklists consist of three sections: “Are the results of the trial valid?” (Section A), “What are the results?” (Section B), and “Will the results help locally?” (Section C). Even though the number of items may be different for each CASP checklist depending on the study design, they allow to compare the methodologies used for each set of answers to one of the three sections. As the CASP checklist does not provide a total score for each study, we chose to classify the studies as either (1) a low-quality study (i.e., participants not recruited in an acceptable way and weak results), (2) a medium-quality study (i.e., participants recruited in an acceptable way and weak to moderate results) or (3) a high-quality study (i.e., participants recruited in an acceptable way and strong results).

## 3. Results 

**Figure 1:**
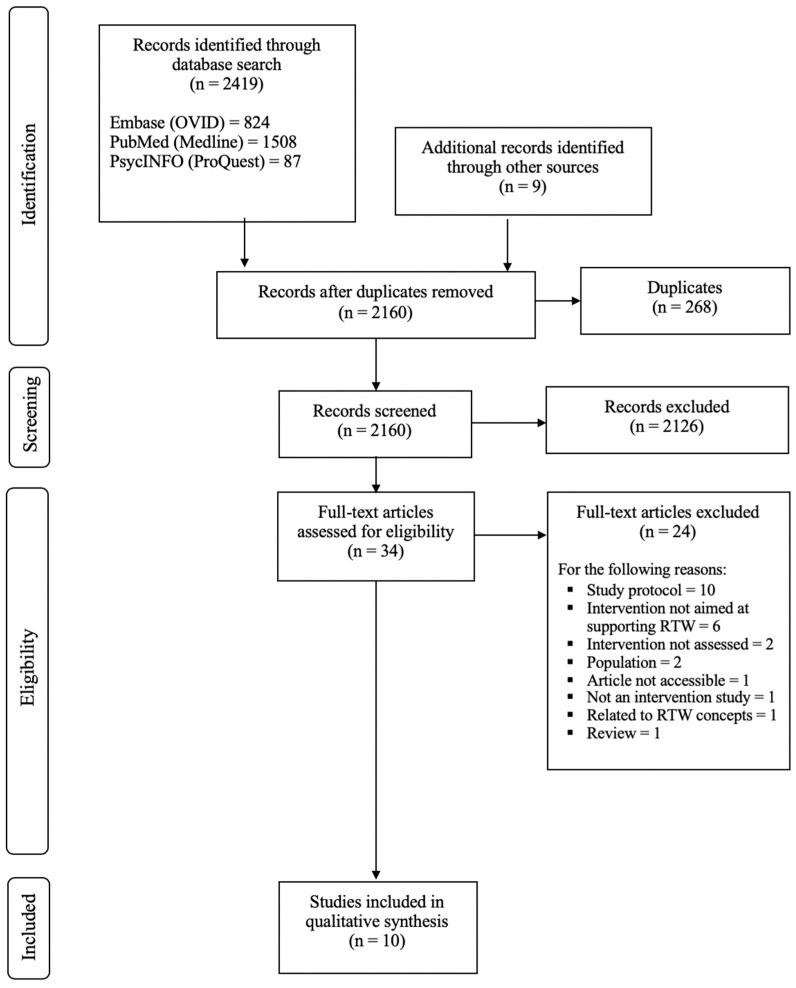
Flow diagram of study selection according to PRISMA.

The initial search returned 2419 records, of which 34 (among them, two articles identified through reference lists and authors’ names) were retained for full-text analysis. Finally, 10 articles [33,34,35,36,37,38,39,40,41,42] were included in this systematic review without disagreement (i.e., inter-judge agreement = 100%). Figure 1 presents a flow-diagram of the research article selection process. 

### 3.1. Study Design and Participant Characteristics

The 10 studies included in the review were published between 2006 and 2017 and conducted in Europe (Netherlands, *n* = 4; United-Kingdom, *n* = 4; Norway, *n* = 2). Three were RCT, three quasi-experimental studies (i.e., pre-post intervention studies), three longitudinal studies and a qualitative study (see Table 1). Among these studies, three are feasibility studies [33,34,35,36].

The majority of the interventions developed in these studies did not specifically address one cancer location (*n* = 7 out of 10; [35,36,37,39,40,41,42]). Nevertheless, more than 50% of the patients recruited in these studies were breast and gynecological cancer patients. Three interventions were specifically adapted for brain [38], breast [34] or colorectal [33] cancer patients. 

Intervention programs were offered to either patients undergoing treatment (*n* = 4 out of 10; [33,34,35,36]), or to patients who had completed primary treatment for at least 2 weeks [39], 1 year [40,42] or for less than 2 years [37] (i.e., chemotherapy and/or radiotherapy) (*n* = 5; [37,39,40,41,42]) or to all the patients, independently of the disease track (*n* = 1 out of 10; [38]). In total, 499 patients (from 7 to 106 patients), mostly women (425 women and 74 men), were included in these studies, with a mean age ranging from 45.8 to 56.25 years. 

### 3.2. Quality Assessment of the Included Studies

The results of the quality assessment are summarized in Table 2 (for more details, see Supplementary Material File S2). Very high inter-judge agreement (93.7%) was obtained. Quasi-experimental studies (*n* = 6; including longitudinal and quasi-experimental designs) were classified in the “low-quality” group because participant recruitment did not fit the eligibility criteria (see answers to Q1 and Q2). All three RCT studies were classified in the “medium-quality” group. RCT study methodologies were strong, although patients and sometimes researchers were aware of the allocation group (see answers to Q4). However, the RCT studies had weak results (see answers to Q7 and Q8) and the benefits of the interventions did not seem worth the workload and costs (see answers to Q11). Finally, the qualitative study included in the review was classified in the “strong-quality” group. However, we cannot answer question Q6 to say whether the relationship between researcher and participants was taken into adequate consideration.

### 3.3. Intervention Theoretical Framework and Program Development

The interventions presented in the included studies were based on a theoretical model (*n* = 5 out of 10; [34,36,39,40,42]), on previous published results (*n* = 5 out of 10; [33,35,39,40,42]) and/or designed with the help of cancer survivors or health professionals (*n* = 5 out of 10; [33,35,39,40,42]). However, in three studies [37,38,41], the authors did not specify how they designed their interventions. 

Theoretical models and theories used to design the interventions differed among all the studies included in this work. Researchers based their interventions on the bio-psycho-social model [34], graded activity (i.e., step by step intervention) and goal-setting theories [36], the self-regulation model and goal-setting theories [39], the shared care model (i.e., the intervention was included in the care pathway) [40] or the attitude-social influence-efficacy theoretical model [42].

Program development is not clear in the included studies. Interventions tested with RCTs were not previously pilot tested [34,40,42]. Interventions tested with quasi-experimental and longitudinal studies do not report program development strategy [33,35,36,37,38,41]. However, three studies specify that they are undergoing a feasibility study [33,35,36] and conclude on the relevance of confirming their results with a RCT. Finally, Schumacher et al. [39] are the only one to describe their program development. They made a feasibility study before to conduct an RCT, and report participants engagement with the intervention and utilization of the content provided in their article.

### 3.4. RTW Interventions

RTW interventions involved cancer patients under or after treatment. These interventions were hospital rehabilitation programs (*n* = 6 out of 10), programs performed outside the hospital (*n* = 3 out of 10) or interventions proposed both at and outside the hospital (*n* = 1 out of 10). The interventions presented in each study are described in Table 3.

#### 3.4.1. Hospital Rehabilitation Programs

Six interventions (out of 10) were hospital rehabilitation programs [34,35,37,38,40,41]. The interventions performed were of different types, potentially adapted or not to the patient’s needs. After initial and individual counselling with a health care professional to identify the patient’s needs and difficulties, patients were referred to adapted services (e.g., social services, psycho-oncology, physician, pain management) [34,38]. Furthermore, in the study described by Rusbridge et al. [38], health care professionals made contact directly with the patient’s employer to suggest specific workload adaptations, create a RTW plan and accompany the patients more closely in RTW.

In Tamminga et al. [40], RTW guidance was provided with a single health care professional for several meetings on RTW, supporting patients, providing patient education, answering their questions and drawing up a RTW plan. In their intervention, health providers also tried to improve physicians’ communication skills to help patients find suitable help.

Moreover, RTW counselling was sometimes performed in parallel with physical training [35,37,41]. In the Oldervoll et al. [37] and Thorsen et al. [41] studies, RTW counselling was concomitant with patient education sessions covering, for example, topics related to cancer treatment, side effects and work situation, nutrition and coping strategies. These sessions were followed by group discussions to allow patients to bring up new subjects. 

Intervention types varied. The intervention was either proposed to inpatients or outpatients, with a full day weekly organization [41] for individual counselling adapted to the patients’ needs, and offered during several months with a limited duration (1 to 15 months) [35,37,38,40,41] or not [34]. 

#### 3.4.2. Interventions Outside the Hospital

Three interventions (out of 10) were rehabilitation programs performed outside the hospital [36,39,42]. Two of them were in the format of tools given to patients when leaving hospital: a work-book (i.e., a leaflet with practical exercises) [39] or a leaflet to help RTW [36]. These guidance tools gave information to patients on RTW (e.g., advices on how drawing up a RTW plan) and symptom management. There was no time limitation in their use. Among these interventions, only Schumacher et al. [39] proposed phone consultation to allow patients to ask questions and discuss the guidance tool given to them. Nieuwenhuijsen et al. [36] gave a more informational tool in the format of a leaflet.

The last kind of intervention proposed outside the hospital was conducted with trained coaches to help patients find a job and guide them when returning to work. In their intervention, van Egmond et al. [42] assessed patients’ readiness to RTW and adapted their guidance to the patients (i.e., helping the patient find a job or become more involved in RTW activities in order to be better prepared to find a job). After 4 months of intervention, if patients were not ready to RTW, they were referred to usual care (i.e., few meetings with an insurance physician and a labor market or re-integration expert to discuss work ability and opportunities for RTW).

#### 3.4.3. Combined Hospital and Outside Hospital Interventions

One intervention (out of 10) combined a hospital rehabilitation program and guidance outside the hospital [33]. This study proposed supporting RTW consultation (i.e., to give advice on RTW) and providing the patient with a leaflet. There was a single RTW consultation at the hospital aimed at providing advice to the patient on his/her treatments and the nature of his/her work (i.e., work ability for manual or non-manual jobs). At the end of this consultation, a leaflet was given, including information on symptoms management, advice on how to talk to the employer and work ability. In Hubbard et al. study [34], patients also received a booklet, but their intervention was essentially a hospital rehabilitation program as this booklet was given during usual care. 

### 3.5. Intervention Effects or Results: Primary and Secondary Outcomes Measured in the Included Studies

The effects of the interventions tested are presented in Table 4. Of the 10 studies, primary outcomes were work related in nine studies (e.g., change in work status, number of days between inclusion in the intervention and RTW) [33,34,35,37,38,39,40,41,42]. Secondary outcomes were: quality of life, fatigue, physical activity, participation in society (e.g., visits to friends and family, housekeeping, outdoor activities) or investigated the association between RTW and sociodemographic or medical variables. In Nieuwenhuijsen et al. [36], the primary outcome was to assess patients’ and physicians’ satisfaction with the intervention and their secondary outcome was to examine the relation between adherence to the intervention and RTW. 

#### 3.5.1. Primary Outcomes: Work-Related Outcome Results

Work status improved significantly between before and after the intervention for five (out of six) pre-post interventions [35,36,37,38,41], as did work ability and RTW self-efficacy [35]. In RCTs [34,40,42], no significant differences in work status was observed, although work status did improve significantly between before and after the intervention [40]. Thorsen et al. [41] investigated other variables influencing RTW. Patients with unimproved work status had a significantly higher proportion of paired relations (i.e., engaged in a relationship) and levels of fatigue compared to patients with improved work status.

#### 3.5.2. Secondary Outcome Results

Regarding the secondary outcomes, improvements in quality of life and fatigue were observed after three pre-post interventions [35,37,41] and was shown to be maintained over time (up to 18 months) in one program [35]. However, these results were observed only in longitudinal studies with no comparison group. In RCTs, no statistical differences were observed between the intervention and the control group (usual care) in quality of life, fatigue and participation in society [34,42]. Despite this, quality of life improved significantly for breast cancer patients after the intervention, compared to usual care, when it was measured with a specific breast cancer quality of life subscale (FACT-B scale) 6 months after the intervention; but the effect was not maintained at 12 months [34]

#### 3.5.3. Qualitative Results

Qualitative investigations were performed only when a tool (i.e., a work-book or a leaflet) was given to the participants. Patients were satisfied with the information provided in these tools [32,37,39]. Interestingly, 67% of participants (*n* = 6) in the Bains et al. study [33] indicated that the tools were given too late (i.e., they should be given prior to the onset of treatment and not when treatments were already started). However, in the Schumacher et al. study [39], cancer survivors thought the work-book should be given at the final treatment. Participants found the tool helpful in creating a RTW plan, allowing them to identify problems and resolve them, as well as talking with employers and reducing anxiety and uncertainty to RTW. 

## 4. Discussion 

The results of the present systematic review show that interventions aimed at maintaining or enhancing RTW for cancer patients are still scarce and have been tested in studies classified as of “low methodology quality”. These interventions, when compared to usual care, do not significantly improve RTW. These disappointing results may find explanation in the design and the methodology followed to build the interventions. Sociodemographic and medical factors associated with lesser cancer patients returning to work are not taken adequately into account in the interventions. Furthermore, employers are generally not involved in the interventions developed, thus questioning the relevance of the interventions found in this systematic review. A methodological critique on the factors to be taken into account in the development of an RTW interventions is suggested below. 

Indeed, strategies to develop an intervention exist. In behavioral treatment development, the ORBIT model developed by Czajkowski et al. [43] is the most recognized in the scientific community as it was developed for use in a broad array of chronic illnesses and uses terminology understandable by different healthcare professionals. According to this model, when designing an intervention, three phases are essential: (1) to identify of a significant clinical question, (2) to define the concepts and design of the intervention (Phase I) and (3) to pilot-test the intervention (Phase II). In the interventions included in our review, no one seems to have followed these steps. This limitation in intervention development seem to be often reported in behavioral interventions. A previous systematic review on psychosocial intervention programs for parents of children with cancer, also reported major limitations on program development and design provided in scientific literature [44].

One major pitfall is the fact that the concept of RTW is not clearly defined in the literature. One definition could be that: RTW is as a proactive approach initiated by the patient or healthcare professionals to maintain work during treatment or to get RTW (full-time, part-time or with adapted work hours) after treatment. With such definition, RTW does not mean “restoring the patient’s former lifestyle”. However, a consensus should be obtained from international experts on RTW, employers and cancer survivors to provide clear basis for RTW researches. 

Several factors should be taken into account when healthcare professionals and researchers were building an RTW intervention. In RTW interventions, medical and sociodemographic factors associated with patient work status are key elements. For example, in Tamminga et al. [9], factors associated with unemployment for thyroid cancer survivors were highlighted: higher age, lower educational level, higher level of fatigue, higher level of anxiety and depression, as well as lower levels of quality of life were associated with unemployment. In Wang et al. [45], factors associated with higher rates of unemployment for breast cancer patients after mastectomy were lower educational level and high psychological job demands. More globally, RTW difficulties are more important in breast cancer, gastrointestinal, nasopharyngeal nervous system cancer and gynecology cancer patients, compared to survivors of blood, prostate and testicular cancers [12]. Thus, RTW in cancer survivors is multifactorial and several factors should be taken into consideration when designing an intervention aiming at supporting RTW and/or should be assessed before. The majority of the studies included in this systematic review offered interventions to patients, regardless of tumor location and medical or sociodemographic variables. This could explain the absence of significative results in RCTs [34,40,42].

In RTW interventions, employers should be involved. In the studies included in the review, the role of employers was not considered. Employers are recognized to play an important role as they can provide support to patients [23,25,46,47]. On the other hand, they can induce negative attitudes such as creating stress for patients [48,49]. Employers lack knowledge on how to retain qualified employees with cancer or chronic diseases, and how to respond appropriately to their needs [25,50]. Thus, not only patients, but also employers need to be accompanied in RTW. Unfortunately, the efficacy of interventions designed to help employers support RTW of sick workers were not performed yet [18]. Results of the intervention studies included in the systematic review may have been related to the health professionals involved to help cancer patients in RTW, such as social workers or occupational therapists (see Table 1). In Morrison and Thomas [22,51], researchers required a holistic, client-centered and collaborative approach to accompany cancer survivors in RTW. In RTW intervention, a multidisciplinary team (including a physician, an occupational therapist, a physiotherapist, a psychologist or psychiatrist, a neuropsychologist and a vocational counselor) would appear to be the most relevant choice to accompany cancer survivors in developing a RTW plan and to advocate the patients’ needs to the employer [51]. 

Furthermore, the majority of the studies included unidimensional interventions (i.e., aimed only at supporting RTW). Multidimensional interventions (e.g., combined physical, psychological and RTW interventions) are more effective in improving RTW [27,36]. Multidimensional RTW interventions are based on a bio-psycho-social model, and in the studies included, only the intervention in Hubbard et al. [34] was based on this model. Four other interventions were based on a theoretical model [36,39,40,42]. The theoretical framework used to design interventions has an important impact. Unfortunately, this was neglected in some of the studies included in this review. The theoretical frameworks of the interventions included in this systematic review were not clear and the RTW interventions proposed varied from informational to more complex programs including counselling and therapeutic patient education. 

Finally, the concept of recovery should be considered when designing an RTW intervention. This concept was initially used in addiction and mental illness [52], but it proves to be well adapted to patients treated for chronic conditions. Recovery means survivors can regain a meaningful life, despite persistent symptoms [53]. Cancer survivors have to manage multiple hurdles after treatment (e.g., late adverse effects, how and when to announce they were diagnosed with cancer, how to manage follow-up exam, physical activity and RTW) [54]. Thus, RTW is one of the components of the survivor’s recovery.

Box 2 **Recommendations to design return to work interventions.**

**1.**  **Define the concept of return to work (RTW):** a systematic review on RTW and how researchers, health agencies and ministries consider or assess RTW should be realized. A work group should be created with international experts on RTW, employers and cancer survivors to define clearly what RTW means after a chronic disease.
**2.**  **Define how to assess RTW:** based on the results found in the literature, the most appropriate way(s) to assess RTW should be defined. Researchers should answer the following question: RTW should be evaluated equally or with different variables from one condition to another?
**3.**  **Explore the literature and take into consideration the appropriate variables:** researchers should target their intervention for a specific population and take into account several variables (e.g., age, tumor location, socioeconomic status) when designing their intervention.
**4.**  **Include appropriate people:** healthcare professionals, employers, patients or survivors, and representatives of health agencies should be included when designing an intervention or in the intervention program.
**5.**  **Write an intervention program manual for professionals:** A detailed manual presenting the steps followed by healthcare professionals when conducting the intervention should be provided. It will allow researchers to communicate easier on their programs and to adapt it to clinical practice.
**6.**  **Pre-test the intervention with few participants, then pilot-test the intervention before to test the efficacy of the program.**

In Box 2, we present a schematic representation of the steps researchers should follow to build an intervention to help patients to RTW. For improved ecological interventions, not only patients but also employers should be considered and included. Based on data of the literature, interventions should be supported by a theoretical model. Step one (1): assess patient fears, needs and willingness to RTW. Step two (2): accompany patients in RTW. The first step could be done at the hospital or outside the hospital, with the help of a guidance tool given to the patients, as suggested in Nieuwenhuijsen et al. [36] and Schumacher et al. [39]. Health professionals should help patients regain self-confidence and design a feasible and personalized RTW plan. When cancer patients or survivors are ready or want to RTW, employers should be included in the intervention. Health professionals could accompany patients through RTW by contacting the employer to adapt work tasks and by providing phone guidance to patients. To date, none of the interventions that have been developed seem to have addressed on the first step: helping patients regain self-confidence and want to RTW. However, patients are at the center of RTW intervention and need to be accompanied from the RTW intention to the action and maintenance of RTW.

This review has certain limitations. First, even though our search was extensive, we cannot be sure that all relevant articles were included. This may explain why no studies were found describing interventions conducted in North America (see Table 1). Secondly, intervention studies published in other sources than peer-reviewed journals were not included. We can suppose that some authors have presented their work in non-scientific journals. A literature review focusing on RTW interventions published in grey literature have therefore be conducted by our team [55]. Thirdly, the results of RTW interventions may not yet have been published. In our systematic review, we excluded protocol-studies. However, during record screening, KL identified five study protocols [56,57,58,59,60] published recently. Researchers should keep a careful watch for the dissemination of the results from these ongoing studies. The interventions presented in these protocols are presented in File S3 of the Supplementary Material. Fourthly, it would have been interesting to contact the authors of the interventions developed and included in this review to have information on how they developed their intervention. Finally, RTW was not assessed with the same measurements in all the studies. We suppose that this is due to the lack of a shared definition for the concept of RTW. Researchers should define clearly what RTW is in severe illness, considering the impact of the disease, type of work (e.g., manual), work ability and elements describing the experience of work following a severe illness such as cancer (self-identity, meaning and significance of work, family and financial context, work performance and environment) [61]. An article dedicated to the definition of RTW is therefore needed.

## 5. Conclusions

This systematic review highlights the difficulties encountered by health professionals and researchers in helping cancer patients RTW. Unidimensional interventions, as included in this review, seem not to be effective in improving RTW and are not well designed from a methodological point of view. To date, no intervention seems to have been effective in helping cancer patients RTW. Six recommendations to design RTW interventions are presented. The most important are to define clearly the concept of RTW and the variables to consider when assessing RTW. Furthermore, healthcare professionals should work in collaboration with employers and national care agencies to create more integrated interventions to help RTW. To design these interventions, a comparison with effective RTW interventions offered to patients suffering from a chronic disease (other than cancer) could be helpful.

## Figures and Tables

**Table 1:** Characteristics of the included studies.

Characteristics	Number	References
*Country of publication*		
Netherlands	4	[35,36,40,42]
United-Kingdom	4	[33,34,38,39]
Norway	2	[37,41]
*Intervention adapted to one cancer type*		
Yes	3	[33,34,38]
No	7	[35,36,37,39,40,41,42]
*Studies design*		
Longitudinal study	3	[35,36,37]
Quasi-experimental study	3	[33,38,41]
Randomized control trial	3	[34,40,42]
Qualitative study	1	[39]
*Participants*		
Women	425	-
Men	74	-
Total	499	-

**Table 2:** The CASP checklists results for assessing the methodological quality of the included studies.

**Cohort Studies**	**Q1**	**Q2**	**?**	**Q3**	**Q4**	**Q5a**	**Q5b**	**Q5a**	**Q6b**	**Q7**	**Q8**	**Q9**	**Q10**	**Q11**	**Q12**
Bains et al. (2011) [33]	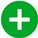	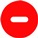	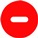	NA	NA	NA	NA	NA	NA	NA	NA	NA	NA	NA	NA
Leesen et al. (2017) [35]	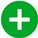	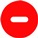	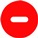	NA	NA	NA	NA	NA	NA	NA	NA	NA	NA	NA	NA
Nieuwenhuijsen et al. (2006) [36]	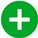	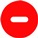	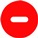	NA	NA	NA	NA	NA	NA	NA	NA	NA	NA	NA	NA
Oldervoll et al. (2014) [37]	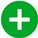	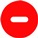	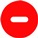	NA	NA	NA	NA	NA	NA	NA	NA	NA	NA	NA	NA
Rusbridge et al. (2013) [38]	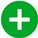	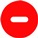	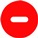	NA	NA	NA	NA	NA	NA	NA	NA	NA	NA	NA	NA
Thorsen et al. (2016) [41]	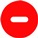	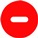	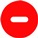	NA	NA	NA	NA	NA	NA	NA	NA	NA	NA	NA	NA
**Randomized controlled trial studies**	Q1	**Q2**	**Q3**	**?**	**Q4**	**Q5**	**Q6**	**Q7**	**Q8**	**Q9**	**Q10**	**Q11**			
Hubbard et al. (2013) [34]	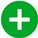	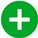	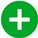	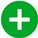	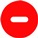	+/−	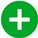	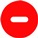	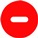	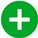	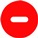	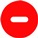			
Tamminga et al., (2013) [40]	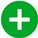	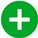	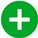	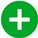	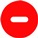	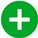	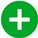	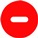	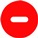	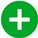	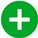	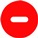			
Van Egmond et al. (2016) [42]	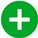	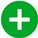	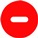	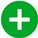	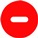	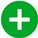	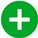	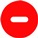	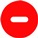	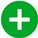	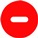	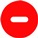			
**Qualitative studies**	**Q1**	**Q2**	**?**	**Q3**	**Q4**	**Q5**	**Q6**	**Q7**	**Q8**	**Q9**	**Q10**				
Schumacher et al. (2017) [39]	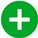	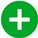	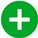	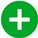	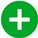	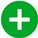	+/−	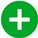	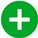	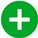	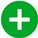				

Question key: Questions for cohort study assessment: Q1 = “Did the study address a clearly focused issue?”, Q2 = “Was the cohort recruited in an acceptable way?”, ? = “Is it worth continuing?”, Q3 = “Was the exposure accurately measured to minimize bias?” Q4 = “Was the outcome accurately measured to minimize bias?”, Q5a = “Have the authors identified all important confounding factors?”, Q5b = “Have they taken account of the confounding factors in the design and/or analysis?”, Q6a = “Was the follow up of subjects complete enough?”, Q6b = “Was the follow up of subjects long enough?”, Q7 = “What are the results of this study?”, Q8 = “How precise are the results?”, Q9 = “Do you believe the results?”, Q10 = “Can the results be applied to the local population?”, Q11 = “Do the results of this study fit with other available evidence?”, Q12 = “What are the implications of this study for practice?”; Questions for randomized controlled trials assessment: Q1 = “Did the trial address a clearly focused issue?”, Q2 = “Was the assignment of patients to treatments randomized?”, Q3 = “Were all of the patients who entered the trial properly accounted for at its conclusion?”, ? = “Is it worth continuing?”, Q4 = “Were patients, health workers and study personnel ‘blind’ to treatment? ”, Q5 = “Were the groups similar at the start of the trial?”, Q6 = “Aside from the experimental intervention, were the groups treated equally? ”, Q7 = “How large was the treatment effect? ”, Q8 = “How precise was the estimate of the treatment effect?”, Q9 = “Can the results be applied to the local population, or in your context? ”, Q10 = “Were all clinically important outcomes considered? ”, Q11 = “Are the benefits worth the harms and costs? ”;. Questions for qualitative studies assessment: Q1 = “Was there a clear statement of the aims of the research?”, Q2 = “Is a qualitative methodology appropriate?”, ? = “Is it worth continuing?”, Q3 = “Was the research design appropriate to address the aims of the research?”, Q4 = “Was the recruitment strategy appropriate to the aims of the research? ”, Q5 = “Was the data collected in a way that addressed the research issue? ”, Q6 = “Has the relationship between researcher and participants been adequately considered? ”, Q7 = “Have ethical issues been taken into consideration? ”, Q8 = “Was the data analysis sufficiently rigorous? ”, Q9 = “Is there a clear statement of findings? ”, Q10 = “How valuable is the research? ”. Answers legend: 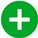 = yes or strong; 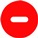 = no or weak; +/− = can’t tell; NA = not administered.

**Table 3:** Description of the interventions presented in the included studies.

[Ref.] Author (year), Country	Cancer Site	Objectives of Intervention	Theoretical Framework	Intervention Methods	Structure of Intervention	Number and Discipline of Trainer or Counsellors
**[33] Bains et al. (2011), United Kingdom**	Colorectal (100%)	To provide work-related advice.	Based on RTW literature and work related guidelines offered by national cancer charitable organizations on information provided to patients.	An individual return to work (RTW) consultation and a ‘Managing Cancer and Employment’ educational leaflet were provided to the participants. The consultation included tailored advice based on the individual’s type of treatment and nature of his/her work (manual/nonmanual). The intervention leaflet was designed to offer information to patients according to whether they were employed in a manual or nonmanual job on the following aspects: managing symptoms at work, communication with employer, and work ability during and after treatment.	One-to-one RTW guidance verbally and in the form of a written educational leaflet.	A researcher provided individual RTW consultation.
**[34] Hubbard et al. (2013), United Kingdom**	Breast (100%)	To assess patients’ needs and to provide adapted support to help them RTW.	Based on the bio-psycho-social model.	First, patients had to complete a questionnaire assessing individual needs to enable RTW, then assessed by phone consultation. Based on this assessment, individuals were referred to relevant services that could support them with cancer-related and treatment side effects. Participants also received a booklet on ‘Work and Cancer’.	One phone consultation with a working health professional followed by a different combination of intervention adapted to the patient’s needs.	Working Health Services.
**[35] Leensen et al. (2017), Netherlands**	Breast (83.9%), colorectal (8.9%), non-Hodgkin’s lymphoma (5.4%) and other localization (2.2%, not specified)	To increase the likelihood of a timely and enduring RTW in cancer patients.	Based on scientific literature and interviews with care providers in the field of occupational health, oncology, sports medicine and physio-therapy.	Before the program, a sports medical assessment was realized. Then, physical training was proposed to the participants (ergometer and resistance exercises of the large muscle groups). Exercises were performed ranging from two series of eight repetitions to three series of 12 repetitions with increasing weight. Alongside the exercise program, participants received 1 to 3 individual counselling sessions on work resumption and work ability.	Twelve weeks of physical training, twice a week for a maximum of 1 hour per session.	An oncology occupational physician, a sport physician and physiotherapist.
**[36] Nieuwenhuijsen et al. (2006), Netherlands**	Breast (50%), male genitals (15%), female genitals (12%), gastro-intestinal (15%) and 12% other	To enhance the communication of information between the patient and the occupational physician on the illness and RTW.	Based upon the principles of graded activity and goal-setting.	First, the letters sent routinely to the general practitioner (on the disease and treatments) were sent to the occupational physician. Then, an educational leaflet containing 10 steps that cancer survivors can undertake to enhance their RTW was given (e.g., draw up an RTW plan).	An education leaflet was given to patients. There was no limited time in its use.	A radiation oncologist.
**[37] Oldervoll et al. (2014), Norway**	Breast (70%) and gynecological (30%)	To reduce drop-out from the work force by providing physical, psychological and social support.	No information provided.	The intervention can be proposed to inpatients and outpatients. The intervention consists in physical exercise, patient education and group discussions. Patient education themes: (1) cancer treatment and its side-effects; (2) physical activity; (3) nutrition; (4) economy and work situation including patient rights within the welfare system; (5) factors that can contribute to a permanent RTW for cancer patients; (6) partnership and sexuality; (7) psychological reactions in relation to cancer; and (8) distress management and coping strategies. These themes were then discussed in group discussions. Patients could also bring new subjects of discussion during these groups.	Inpatient program: Four weeks (three weeks stay and one more week 8 to 12 weeks later to increase patient’s motivation) Outpatient program: seven weeks (5 hours per day) Every day, patients had physical exercises to perform, patient education and group discussions.	At least one social worker, one health professional, one physiotherapist and one sport instructor.
**[38] Rusbridge et al. (2013), United Kingdom**	Brain (100%)	To support patients to overcome the barriers faced when returning to work or remaining in work.	No information provided.	Support was adapted to patients needs after an initial assessment of patients’ impairment and job demand to establish short and long-term goals. Interventions took the form of patient-based symptom management (e.g., fatigue, relaxation) and workplace intervention (e.g., scheduling, strategies to manage memory impairment). A professional contacted the employer to suggest specific workload adaptations, created RTW plan and accompany patients to adopt strategies adapted to their work.	An average of 11 hours sessions in 5 months	An occupational therapist and a neuropsychologist.
**[39] Schumacher et al. (2017), United Kingdom**	Breast (52%), urological (30%), bowel (13%) and gynecological (4%)	To support RTW.	Based on self-regulation model and goal-setting theories and scientific literature.	A work-book was given to the patients. It was composed of 4 chapters and included activities to encourage thoughts/beliefs about cancer and how it could affect work, develop goals around RTW with small achievable steps, culminating in the creation of a RTW plan. Two support phone consultations at week 2 and 4 gave participants the opportunity to discuss their progress, ask questions about items they found difficult, and seek clarification on any of the workbook content.	Four weeks (but there was no real limit time in its use).	Not specified.
**[40] Tamminga et al., (2013), Netherlands**	Breast, (64%) cervix (23%), ovarian (5%), vulva (3%) and other (5%, not specified)	To support RTW and improve quality of life.	Based on the shared care model, scientific literature and interview with both cancer survivors and professionals.	The intervention was composed of three steps: (1) delivering patient education and support at the hospital, as part of usual psycho-oncology care (4 meetings of 15 minutes); (2) improving communication between the treating physician and the occupational physician by sending at least one letter; (3) drawing-up a RTW plan in collaboration with the employer.	Maximum of 14 months	An occupational physician, an oncology nurse and a medical social worker.
**[41] Thorsen et al. (2016), Norway**	Breast (60%), gynecological (31%), lymphoma (7%) and esophagus (1%)	To improve work ability and health related quality of life.	The program was initiated by the Norwegian government. However, no information on intervention design and theoretical framework are provided.	At the start and end of the program, each patient had a consultation with a social worker focusing on individual goals for the program period. Each day, the program started with a patient education session for 2 h. These sessions covered topics related to cancer treatment, side effects, partnership and sexuality, economy and work situation, nutrition, physical exercise and coping strategies. The patient education was followed by 1-h group discussion of the topic presented. After lunch the participants performed physical activity (e.g., Nordic walking, water gymnastics, yoga) for 60–120 min.	Full day weekly for 7 weeks	A least one social worker, one physiotherapist, one nurse and one physician (a radiotherapist).
**[42] van Egmond et al. (2016), Netherlands**	Breast (39.8%), lung (1.8%), gynecological (4.1%), colon (7.6%), gastro-intestinal (5.8%), head and neck (4.7%), prostate (1.8%), hematological (13.5%), brain (4.7%) and other type of cancer (14%, not specified)	To support RTW after job loss.	Based on the attitude-social influence-efficacy theoretical model, scientific literature and focus groups with cancer survivors.	First, an introductory interview was conducted to discuss RTW plans and assess whether the patients were actively involved in looking for jobs or not involved in RTW activities. Then, patients were allocated to one of the following routes. - Route 1: If patients were involved in RTW activities, participants were placed in therapeutic or paid work with the support of two job hunting agencies. - Route 2: If patients were not involved in RTW activities, participants were helped to RTW (e.g., creation of a RTW plan) and coached on several themes (e.g., fatigue management, communication about cancer). The participants motivation to RTW was assessed after 4 session of coaching and several sessions later if the patients was not ready to RTW. If patients were ready to RTW, the intervention continued with route 1. If they were not ready to RTW after the intervention, they were referred to usual care.	Maximum of 4 months.	Job hunting agencies and re-integration agency (coaches).

**Table 4:** Results of the interventions and outcomes measured in the included studies.

[Ref.] Author (year), Country	Intervention Group		Control Group		Primary Outcome	Secondary Outcome	Follow-up Assessment	Main Results
	*N*, *n* Female (%), *n* Male (%)	Socio-demographic Data (Age, Education, work Status)	*N, n* Female (%), *n* Male (%)	Socio-Demographic Data (Age, Education, Work Status)				
[33] Bains et al. (2011), United Kingdom	*N* = 13, female *n* = 5 (39%), male *n* = 8 (61%)	Mean age: 56.25 years (SD, 5,75) Level of education: low (23%) intermediate, (38.5%), high (38.5%) Work status: on sick leave (31%), continued to work during treatments (69%)	/	/	Current sickness leave status, return to work (RTW) intentions and perceived work ability	/	T0 (baseline) and T1 (6 months)	No significant effect of the intervention on work ability, self-efficacy, anxiety, and depressive symptoms. Nine patients found the intervention ‘useful’ or ‘very useful’.
[34] Hubbard et al. (2013), United Kingdom	*N* = 7 female	Mean age: 49.7 years (SD, 7.6) Level of education: NA Work status: full time (85.7%), part time (14.3%)	N=11 female	Mean age: 49.7 years (SD, 7.6) Level of education: NA Work status: full time (45.5%), part time (54.5%)	Self-reported sickness absence	Change in employment pattern, health related quality of life and fatigue	T1 (6 months) and T2 (12 months)	No significant differences observed on primary and secondary outcomes.
[35] Leensen et al. (2017), Netherlands	*N* = 93, female *n* = 84 (90.3%), male *n* = 9 (9.7%),	Mean age: 47.9 years (SD, 7.4) Level of education: low (14%), intermediate (33.3%) and high (52.7) Work status: on sick leave (100%)	/	/	Time to RTW between first date of sick leave and the first date of resumption	Perceptions regarding work (importance of work, work ability, self-efficacy regarding RTW and work limitations), physical factors (muscle strength, cardiorespiratory fitness, physical activity level and fatigue) and quality of life.	T0 (baseline), T1 (6 months), T2 (12 months) and T3 (18 months)	Regarding RTW: 59% of the participants RTW at T1, 86% at T2 and 83% at T3. Significant improvements (*p* < 0.05) were observed in the importance of work, work ability, RTW self-efficacy, fatigue and quality of life.
[36] Nieuwenhuijsen et al. (2006), Netherlands	*N* = 26, gemale *n* = 19 (73%), male *n* = 7 (27%)	Mean age: 45.8 years (SD, 6.5) Level of education: NA Work status: NA	/	/	Patients’ and occupational physicians’ satisfaction with the intervention	To examine the relation between adherence to the advice and RTW.	Not clear. RTW assessed at 6, 12 and 18 months	The leaflet was perceived as useful (score of 7 on 10). Regarding RTW: 65% of the participants RTW at 6 months, 89% at 12 months and 92% at 18 months. Level of adherence to the program is not significantly related to RTW.
[37] Oldervoll et al. (2014), Norway	*N* = 56 female	Inpatient program Mean age: 51 years Level of education: low to intermediary (44%), high (56%) Work status: full time (14%), part time (20%), sick-leave (66%)	*N* = 60 female	Outpatient program Mean age: 50 years Level of education: low to intermediary (55%), high (45%) Work status: full time (23%), part time (10%), sick-leave (67%)	Change in work status	Fatigue and health related quality of life	T0 (baseline), T1 (after the intervention) and T2 (6 months later)	In Inpatient group, 73% of patients on sick-leave or in part-time work improved their work status after the intervention. In Outpatient group, 76% of patients on sick-leave or in part-time work improved their work status after the intervention. There were no statistical differences between the two groups. Fatigue and health-related quality of life improved significantly between T0 and T2 in the two groups, but no statistical differences were observed between the groups.
[38] Rusbridge et al. (2013), United Kingdom	*N* = 34, female *n* = 15 (41%), male *n* = 19 (59%)	Mean age: 46 years (SD, 11) Level of education: low to intermediary (41%) and high (59%) Work status: working (32%) and not working (68%)	/	/	work status	Relations between work status after the intervention and demographic and tumor-related factors	T0 (baseline) and T1 (discharge)	More patients RTW after the intervention (*p* < 0.05). Furthermore, physical disability decreased the likelihood of RTW.
[39] Schumacher et al. (2017), United Kingdom	*N* = 23, female *n* = 16 (70%), male *n* = 7 (30%)	Mean age: 50 years Level of education: low to intermediary (41%) and high (59%) Work status: working (100%)	/	/	Work-related outcomes and utilization of the intervention	/	Interview four weeks after the intervention	Participants observed changes in their empowerment. The RTW plan was perceived as helpful to identify problems and solutions, but also to discuss with employers. Patients felt less anxious and uncertain about RTW. Patients thought the intervention should be conducted during the sole or final treatment.
[40] Tamminga et al., (2013), Netherlands	*N* = 56 female	*N* = 65 at the beginning of the intervention Mean age: 47.5 years (SD, 8.2) Level of education: low (11%), intermediary (59%) and high (30%) Work status: on sick leave (100%)	*N* = 59 female	*N* = 68 at the beginning of the study Mean age: 47.6 years (SD, 7.8) Level of education: low (16%), intermediary (51%) and high (33%) Work status: on sick leave (100%)	RTW and quality of life	Work ability, work productivity and cost	T0 (baseline), T1 (6 months) and T2 (12 months)	Regarding RTW: 79% of the participants RTW in both groups at 12 months (*p* < 0.05) and quality of life improved significantly over time but did not differ statistically between groups. Work ability improved significantly over time but did not differ significantly between groups. Work functioning did not improve significantly over time and did not differ significantly between groups. The costs did not differ statistically between groups.
[41] Thorsen et al. (2016), Norway	*N* = 106 female	Mean age: 48.82 years Level of education: low and intermediary (40.57%), high (59.43%) Work status: full time (9%), part time (9%), on sick-leave (76%), Work assessment allowance (5%)	/	/	To identify the proportion of female patients with unimproved work status 6 months after termination of the program	To identify demographic-, disease- and health-related characteristics at baseline associated with unimproved work status at follow-up. A third aim was to measure changes in health-related quality of life, fatigue and physical activity after completing the R-RTW program for patients with unimproved and improved work status.	T0 (baseline) and T1 (6 months after the program)	Regarding RTW: 64% of the participants had an improvement of their work status 6 months after the intervention. Participants with unimproved work status had a significantly higher proportion of paired relations and levels of fatigue compared to patients with improved work status. No significant differences were observed on other sociodemographic variables between unimproved and improved work status after the intervention. Health-related quality of life scores increased significantly after the intervention, for both women in unimproved and improved work status groups.
[42] van Egmond et al. (2016), Netherlands	*N* = 85 at inclusion, female *n* = 61 (71.8%), male *n* = 24 (28.2%)	Mean age: 47.9 years (SD, 8.5) Level of education: low (14.1%), intermediary (58.8%) and high (27.1%) Work status: unemployed (100%)	*N* = 86 at inclusion, female *n* = 57 (66.3%), male *n* = 829 (33.7%)	Mean age: 48.8 years (SD, 8.7) Level of education: low (20.9%), intermediary (44.2%) and high (34.9%) Work status: unemployed (100%)	Number of days between the day of inclusion and the first day of sustainable RTW	Rate of RTW per group, fatigue, quality of life and participation in society	T0 (baseline), T1 (3 months), T2 (6 months) and T3 (12 months)	No significant differences between the groups on the variables measured were observed.

## Supplementary Materials

The following are available online at https://www.mdpi.com/1660-4601/16/8/1343/s1, File S1: Research equations used in the databases, File S2: Complete details of the quality assessment of the studies included, File S3: Presentation of the interventions (*n* = 5) found in study protocols published in scientific journals.
