# Supplementary material for: Return to Work Interventions for Cancer Survivors: A Systematic Review and a Methodological Critique
Source: Int J Environ Res Public Health. 2019 Apr 14;16(8):1343. doi: 10.3390/ijerph16081343 (PMC6518012; doi:10.3390/ijerph16081343)
Supplement: Supplementary file 1 [file ijerph-16-01343-s001.pdf]

File S1. Research equations used in the databases.

PubMed (31.03.2018)

(cancer) AND (return-to-work OR return to work/organization and administration [MeSH] OR return to work/statistics and numerical data [MeSH] OR re-integrating OR back to work OR employment [MeSH] OR employment sector OR sick leave [MeSH] OR absenteeism [MeSH] OR occupational medicine[MeSH] OR occupational health [MeSH] OR occupational health services [MeSH] OR "disability management" OR "disability prevention" OR employer*) AND (rehabilitation [MeSH] OR rehabilitation program OR training program* OR training tool* OR training OR occupational rehabilitation OR occupational intervention OR workplace intervention OR occupational therapy OR stress management OR work ability) AND (randomized controlled trial [MeSH] OR randomized controlled trial OR controlled clinical trial OR controlled clinical trial [Publication Type] OR evaluation study OR evaluate* OR effects OR effectiveness OR efficiency OR process OR outcome)

Embase (01.04.2018)

(cancer.mp. or exp malignant neoplasm/) and (return to work.mp. or exp return to work/ or re-integrating.mp. or back to work.mp. or exp employment/ or employment.mp. or employment sector.mp. or sick leave.mp. or exp medical leave/ or occupational medicine.mp. or exp occupational medicine/ or occupational health.mp. or exp occupational health/ or occupational health services.mp. or exp occupational health service/ or disability management.mp. or disability prevention.mp. or employer*.mp.) and (rehabilitation.mp. or exp rehabilitation/ or rehabilitation program.mp. or rehabilitation program*.mp. or exp training/ or training program*.mp. or training tool*.mp. or occupational rehabilitation.mp. or exp vocational rehabilitation/ or occupational intervention.mp. or workplace intervention.mp. or occupational therapy.mp. or exp occupational therapy/ or stress management.mp. or exp stress management/ or work ability.mp.) and (randomized controlled trial/ or controlled clinical trial.mp. or exp controlled clinical trial/ or evaluation study.mp. or exp evaluation study/ or evaluate*.mp. or effects.mp. or effectiveness.mp. or efficiency.mp. or process.mp. or outcome.mp.)

PsycInfo (02.04.2018)

IF,TI,AB(cancer) AND (IF,TI,AB(return to work OR reintegrating OR back to work OR employment OR employment sector OR sick leave OR absenteeism OR occupational medicine OR occupational health OR occupational health services OR "disability management" OR "disability prevention" OR employer*) AND peer(yes)) AND IF,TI,AB(return to work or re-integrating or back to work or employment or employment sector or sick leave or absenteeism or occupational medicine or occupational health or occupational health services or "disability management" or "disability prevention" or employer*) AND IF,TI,AB(rehabilitation OR rehabilitation program OR training program* OR training OR training tool* OR occupational rehabilitation OR occupational intervention OR workplace intervention OR occupational therapy OR stress management OR work ability) AND IF,TI,AB(randomized controlled trial OR controlled clinical trial OR evaluation study OR evaluate* OR effects OR effectiveness OR efficiency OR process OR outcome

File S2. Complete details of the quality assessment of the studies included.

Cohort studies	**[33] Bains et al. (2011), United Kingdom**	**[375 Leensen et al. (2017), Netherlands**	**[36] Nieuwenhuijsen et al. (2006), Netherlands**	**[37] Oldervoll et al. (2014), Norway**	**[38] Rusbridge et al. (2013), United Kingdom**	**[41] Thorsen et al. (2016), Norway**
**Section A: Are the results of the study valid? (yes/no/can't tell)**						
1. Did the study address a clearly focused issue?	yes	yes	yes	yes	yes	no
Comments	The authors aim to assess the delivery and format of an return to work (RTW) intervention for colorectal cancer patients. Outcomes measured are specified in the article. However,	The authors aim to assess RTW after an RTW intervention for cancer patients and the changes in several variables (work ability, self-efficacy, work limitations, muscle strength, physical fitness,	The authors aim to assess the delivery and format of a RTW intervention for cancer patients and their satisfaction with the intervention, as well as physicians' satisfaction with the intervention.	The authors aim to assess RTW after an RTW intervention for cancer patients and the changes in several variables (physical fatigue and quality of life) between before and after the intervention.	The authors aim to assess RTW after an RTW intervention for patients with brain tumors. Work status at referral and at discharge from the service was studied, as well as the links between work	The study objective is not clear at first read. However, we understand that the authors aim to assess RTW after an RTW intervention for female cancer patients. Percentage of

	the reason for assessing psychological variables is not clear.	fatigue levels and quality of life) between before and after the intervention.			status after the intervention and demographic and tumor-related factors.	unimproved work status was studied, as well as the links between work status after the intervention and demographic, disease, health-related characteristics, quality of life, fatigue and physical activity.
2. Was the cohort recruited in an acceptable way?	no	no	no	no	no	no
Comments	Only 13 patients were included in the study (n=11 for pre and post intervention evaluation). Reasons for patient exclusion were not presented.	Patients who were unable to perform physical activity were not included in the study as the intervention tested proposed a physical activity. However, regarding	Only 35 patients were included in the study in 8 months. The intervention was proposed to several cancer patients, without considering the location of the	The study was not proposed to all the patients and the reasons are not specified. Also, differences exist between the groups before the start of the intervention	All brain tumors are not represented in the sample. Furthermore, the study was proposed to all the patients in one hospital, at any point in their	Authors state that the study "might include a self-selected sample" and the study was proposed to all the patients in one hospital. The participants

	Furthermore, the study was proposed to only 22 patients in 3 months.	participant characteristics, a majority of breast cancer patients took part in the study and few colorectal and non-Hodgkin lymphoma patients. The authors do not specify this choice and do not present more specifically the characteristics of the participants in terms of cancer location. This represents a major limitation for representativeness of the general population.	tumor. The recruitment was performed in one hospital.	(cancer type and mean number of months since diagnosis). This can compromise the extent to which the findings can be generalized.	disease pathway. This can compromise the extent to which the findings can be generalized. Clear information on participant recruitment is not presented.	might therefore not have been recruited in an acceptable way and this can compromise the extent to which the findings can be generalized.
**Is it worth continuing? (yes/no)**	**no**	**no**	**no**	**no**	**no**	**no**

3. Was the exposure accurately measured to minimize bias?	/	/	/	/	/	/
Comments						
4. Was the outcome accurately measured to minimize bias?	/	/	/	/	/	/
Comments						
5. (a) Have the authors identified all important confounding factors?	/	/	/	/	/	/
Comments						
5. (b) Have they taken account of the confounding factors in the design and/or analysis?	/	/	/	/	/	/
Comments						
6. (a) Was the follow up of subjects complete enough?	/	/	/	/	/	/
6. (b) Was the follow up of subjects long enough?	/	/	/	/	/	/
Comments						

**Section B: What are the results?**						
7. What are the results of this study?	/	/	/	/	/	/
Comments						
8. How precise are the results?	/	/	/	/	/	/
Comments						
9. Do you believe the results?	/	/	/	/	/	/
Comments						

**Section C: Will the results help locally?**						
10. Can the results be applied to the local population?	/	/	/	/	/	/
Comments						
11. Do the results of this study fit with other available evidence?	/	/	/	/	/	/
Comments						

12. What are the implications of this study for practice?	/	/	/	/	/	/
Comments						

Qualitative studies	**[39] Schumacher et al. (2017), United Kingdom**
**Section A: Are the results valid?**	
1. Was there a clear statement of the aims of the research?	yes
Comments	Authors aimed to explore how participants used the workbook aimed at improving RTW in cancer survivors and how participants were engaged with the intervention and utilized the content of the workbook. This study is important and relevant as there is little research examining how participants engage with an intervention in terms of the application or implementation of the material in relation to their individual situations.
2. Is a qualitative methodology appropriate?	yes
Comments	A "framework" analysis approach was used for data analysis and performed independently by two researchers.
**Is it worth continuing? (yes/no)**	**yes**

3. Was the research design appropriate to address the aims of the research?	yes
Comments	In the introduction section, the researchers stated why qualitative research was appropriate and clearly explained their methodology.
4. Was the recruitment strategy appropriate to the aims of the research?	yes
Comments	Participants were recruited from a larger sample of a previous study. Twenty participants were interviewed, allowing data saturation for qualitative studies.
5. Was the data collected in a way that addressed the research issue?	yes
Comments	Themes explored during the interview were relevant to address the research issue, discussed with a research team of health professionals and based on literature review findings. Interview schedule was also pre-tested.
6. Has the relationship between researcher and participants been adequately considered?	can't tell
Comments	The authors do not provide information on their own role in and influence on the data collection and data analysis. However, it is difficult in a scientific journal to determine their theoretical background and their influence. Generally, in qualitative research, the relationship between a researcher and the participants is considered, but not necessarily presented in the publication.

**Section B: What are the results?**	

7. Have ethical issues been taken into consideration?	yes
Comments	Ethical approval was obtained for the study and authors specified in the methodology how the study was presented to the participants.
8. Was the data analysis sufficiently rigorous?	yes
Comments	The results were analyzed independently by two reviewers and sufficient verbatims are presented in the results section. However, no information was presented on the researchers' roles.
9. Is there a clear statement of findings?	yes
Comments	The results were analyzed independently by two reviewers and were discussed in relation with previous research.

**Section C: Will the results help locally?**	
10. How valuable is the research?	yes
Comments	The authors explain why the results are important (e.g., first study to explain how a tool aimed at RTW is used and can facilitate RTW). Future research is presented.

Randomized control trials	**[34] Hubbard et al. (2013), United Kingdom**	**[40] Tamminga et al., (2013), Netherlands**	**[42] van Egmond et al. (2016), Netherlands**
**Section A: Are the results of the trial valid? (yes/no/can't tell)**			

1. Did the trial address a clearly focused issue?	yes	yes	yes
Comments	The authors expected breast cancer patients referred to the intervention (vocational rehabilitation) to experience fewer days off work due to sickness in the first 6 months post-surgery, lower levels of fatigue and increased quality of life compared to patients in the usual care.	The authors wanted to determine the effect of a hospital-based work support intervention (intervention) for cancer patients on RTW and on quality of life, compared to the usual care.	The authors expected offering a RTW intervention to cancer patients to lead to an improvement in duration until RTW, compared to the usual care.
2. Was the assignment of patients to treatments randomized?	yes	yes	yes
Comments	Allocation ratio was 1:1 for the intervention and usual care arms. The randomization procedure was partially blind: a statistician provided the allocation sequences to a researcher; another researcher, who was not aware of participant allocation, was responsible for participant recruitment and data collection.	The ALEA computerized randomization program was used to assign participants to one of the groups.	Participants were randomized in 3 strata considering work status and then they were randomly assigned to one group.
3. Were all of the patients who entered the trial properly accounted for at its conclusion?	yes	yes	no

Comments	A flow diagram is presented in the article and provides a clear explanation of patient exclusion before randomization and exclusion from the analysis.	A flow diagram is presented in the article and provides a clear explanation of patient exclusion before randomization and exclusion from the analysis.	The loss of participants between T1 and T3 is not explained. However, the analyses are performed well.
**Is it worth continuing? (yes/no)**	**Yes**	**Yes**	**Yes**
4. Were patients, health workers and study personnel 'blind' to treatment?	no	no	no
Comments	Participants were aware of their allocation group (it could not be dissimulated). The randomization procedure was partially blind (i.e. the researcher who performed the participant recruitment and data collection was not aware of the participants' group allocation).	Patients and researchers were aware of the allocation as it was impossible to conceal allocation for this study.	Participants were aware of their allocation group (it was impossible to conceal).
5. Were the groups similar at the start of the trial?	can't tell	yes	yes
Comments	Statistical analysis is not provided to determine group similarity before the start of the intervention. However, descriptively, some	No statistical differences were observed between the two groups in	No statistical differences were observed between the two groups in terms of participant characteristics (see Table 1),

	differences were observed between the groups (e.g. in the intervention group, 85.7% were in full-time employment while they were 45.5% in the usual care group).	terms of participant characteristics (see Table 1).	except for 4 variables (ethnicity, more patients with brain tumors in the control group, patients in the control group received more radiotherapy and had a higher level of fatigue).
6. Aside from the experimental intervention, were the groups treated equally?	yes	yes	yes
Comments	The study was presented the same way for all the participants, they completed the same questionnaires longitudinally and both groups received an information booklet.	The study was presented the same way for all the participants and they completed the same questionnaires longitudinally.	The study was presented the same way for all the participants and they completed the same questionnaires longitudinally.

**Section B: What are the results? (strong/moderate/weak/can't tell)**			
7. How large was the treatment effect?	weak	weak	weak
Comments	No statistical difference was observed in the primary and secondary outcomes (except for 1 sub-score - FACT-B BCS).	No statistical difference was observed in the primary and	No statistical difference was observed in the primary and secondary outcomes between the groups.

		secondary outcomes between the groups.	
8. How precise was the estimate of the treatment effect?	weak	weak	weak
Comments	Confidence limits are in a high range.	Confidence limits and median time provided when applicable are in a high range.	Confidence limits are in a high range.

**Section C: Will the results help locally? (yes/no/can't tell)**			
9. Can the results be applied to the local population, or in your context?	yes	yes	yes
Comments	Although the groups were small, participants were representative of the general population.	The results can be applied to the general population (mostly breast and gynecological cancer).	The results can be applied to the general population (mostly breast and hematological cancer).
10. Were all clinically important outcomes considered?	no	yes	no
Comments	Medical information (i.e., cancer stage, co-morbidities) was measured but not used in the statistical analysis. Furthermore,	No statistical differences were observed between the groups,	Medical information (i.e., cancer type, treatments) was measured but not used in the statistical

	participant surgery type (breast conserving surgery or mastectomy) was not specified and can be a factor of work absence duration.	considering the medical variables.	analysis. Furthermore, co-morbidities were not measured.
11. Are the benefits worth the harms and costs?	no	no	no
Comments	No benefits were observed from the intervention compared to the usual care.	No benefits were observed from the intervention compared to the usual care.	No benefits were observed from the intervention compared to the usual care.

File S3. Presentation of the interventions (n=5) found in study protocols published in scientific journals.

Author (year), country	Objectives of intervention	Intervention methods	Structure of intervention	Implementation
[568] Munir et al. (2013), United Kingdom	To help patients manage their work or return to work (RTW) effectively, manage the impact of their cancer-related health on their work, and manage the impact of work conditions upon their cancer related health.	A work-related guidance tool was developed (40 questions to help patients communicate with healthcare professionals or employer). The questions are linked to health, work, finance and indicate to which person to talk to get information on these points.	A work-related guidance tool was given to patients. There was no limited time for its use.	Outside hospital
[579] Stapelfeldt et al. (2015), Denmark	To help RTW	First, patients were asked to complete an online questionnaire to assess patients' readiness for RTW and need for support in order to set up an individual RTW plan. The intervention was guided by the Acceptance and Commitment Therapy (ACT) and the Individual Placement and Support Model. Meetings were set according to the RTW plan.	Maximum of one year	Hospital
[5860] Tamminga et al., (2016), Netherlands	To help RTW	Development of an e-health intervention. The care provider will (1) answer questions, (2) monitor and supervise use of the Cancer@Work intervention, (3) provide personal feedback on assignments of the Cancer@Work intervention and (4) encourage patients to comply with the intervention.	Follow-up of 12 months	Hospital and outside hospital

		To be able to blend their care with the Cancer@Work intervention, care providers had access to a special section of the e-health intervention with which they are able to see whether patients have used the Cancer@Work intervention, see which functionalities each patient has used, evaluate the content of some of the assignments, answer questions from patients, send messages to patients and receive support from and answers to questions from an oncological occupational physician.<br>The Cancer@Work intervention includes: (1) a library to inform patients and various subjects related to RTW (e.g. work adjustment, legal and insurance issues), (2)<br>action to help patients drawing-up a RTW plan, to take action on the potential financial consequences and toward their obligations to social security. Patients can also learn from other patients' experience through frequently asked and answered questions or advices.Patients can also send private messages to their personal care provider, through the e-health tool.		
[5961] Wienert et al., (2016), Germany	Focus on work, work ability and RTW	Patients received conventional medical rehabilitation and work-related medical rehabilitation.<br>Medical rehabilitation included: exercise therapy, physiotherapy, social counseling, occupational therapy, psychological seminars and counseling and	100 hours of therapy maximum	Hospital

		dieting (3 weeks).<br>The work-related medical rehabilitation was composed of 6 modules: additional work related diagnostics, multi professionals team meetings (i.e., individual case conference to discuss patients individual RTW program), introductory session, work-related functional capacity training, work-related psychological groups and intensified social counseling.		
[60~~2~~] Zaman et al., (2016), Netherlands	To help RTW	Support provided to the patient was determined by a questionnaire assessing patients' needs. Three individual meetings with a healthcare professional were provided:<br>1) Inform patients about the importance of work during and after treatment, to identify any work-related problems, and to make a plan for the RTW<br>2) inform and evaluate the goals of the first meeting (3 to 6 months after)<br>3) inform and evaluate the goals of the first and second meetings (6 to 9 months after treatments)	6 to 15 months. Each meeting last around 30 min.	Hospital
